# Endoscopic Innovation Treatment Strategy in Hepatocellular Carcinoma (HCC) Patients Before Immunotherapy: A Case Series Study in Unselected Patients

**DOI:** 10.1002/jgh3.70367

**Published:** 2026-03-07

**Authors:** Stephen Dario Syofyan, Kenoah Kovara, Nicholas Putra Lesmana, Josia Nathanael Wiradikarta, Joshua Francisco Syofyan, Juan Benedict Widjaja, Sri Inggriani, Rio Hermawan, Cosmas Rinaldi Adithya Lesmana

**Affiliations:** ^1^ Faculty of Medicine Universitas Indonesia Jakarta Indonesia; ^2^ Faculty of Medicine Universitas Udayana Denpasar Bali Indonesia; ^3^ Gastrointestinal Cancer Center, MRCCC Siloam Semanggi Hospital Jakarta Indonesia; ^4^ Digestive Disease & GI Oncology Center, Medistra Hospital Jakarta Indonesia; ^5^ Radiology Department Dharmais National Cancer Hospital Jakarta Indonesia; ^6^ Hepatobiliary Division, Department of Internal Medicine Dr. Cipto Mangunkusumo National General Hospital, Medical Faculty Universitas Indonesia Jakarta Indonesia

**Keywords:** case series, endoscopic ultrasound, hepatocellular carcinoma, immunotherapy, variceal bleeding

## Abstract

Hepatocellular carcinoma remains a major problem in Asia as well as globally. Most patients present at the late stage of the disease. Currently, several types of immunotherapy (IT) have been studied for its efficacy in treating hepatocellular carcinoma, such as atezolizumab and bevacizumab, which work as inhibitors in checkpoints and inhibitors in VEGF, respectively. In recent studies, this specific combination of therapy has shown a better progression free survival (PFS) rate and overall survival (OS) compared to the current therapy sorafenib. However, this treatment modality carries several potential adverse effects, such as gastrointestinal bleeding and bleeding esophageal varices (BOV). We present a series of seven HCC patients, where six patients had esophageal varices and one patient had large gastroesophageal varices who underwent endoscopy evaluation and therapeutic endoscopy including endoscopic ultrasound guided before IT. All patients underwent IT after therapeutic endoscopy within less than a week. No bleeding was observed during and after IT. These cases demonstrate the endoscopic ultrasound method in HCC patients.

## 1 Introduction

Hepatocellular carcinoma (HCC) is one of the types of cancer with the 6th highest number of patients according to the Globocan 2020 [1]. HCC often develops in patients with prior liver diseases, such as viral hepatitis infection, metabolic dysfunction‐associated fatty liver disease (MAFLD), alcoholic liver disease (ALD), and aflatoxins [2, 3, 4]. Currently, several types of immunotherapy (IT) have been studied for their efficacy in treating HCC, such as atezolizumab and bevacizumab, which work as inhibitors of checkpoints and inhibitors of VEGF, respectively [5, 6]. In recent studies, this specific combination of therapy has shown a better progression‐free survival (PFS) rate and overall survival (OS) compared to the current therapy sorafenib [7]. But, this treatment modality carries several potential adverse effects, such as gastrointestinal bleeding and bleeding esophageal varices (BOV), where BOV was reported in 103 out of 2062 patients who underwent IT [8]. However, there is still a lack of data regarding the bleeding incidence after IT, and there is no consensus yet regarding the optimal timing to start IT after endoscopic therapy in patients with large gastro‐esophageal varices.

## 2 Case Series

From the 17 consecutive patients who underwent Atezolizumab and Bevacizumab treatment within 1‐year period (Table 1), 6 patients did not undergo the esophagogastroduodenoscopy (EGD) procedure based on Baveno VII criteria. Large esophageal varices (EV) were diagnosed based on Baveno VII (more than 5 mm with or without red sign). The high‐risk varices were also diagnosed based on high‐risk stigmata, such as hematocystic spots. The gastroesophageal varices (GOV) were classified based on Sarin's classification. In the remaining 11 patients who underwent OGD procedure, 4 patients (23.53%) did not have varices while large EV were found in five patients and large GOV were found in one patient. In another patient, only small EV were found. Endoscopic Ultrasound (EUS) evaluation followed by endoscopic band ligation (EBL) was performed in one patient with large EV. A patient with large GOV underwent EUS‐guided cyanoacrylate injection. All patients underwent immunotherapy within less than a week after therapeutic endoscopic intervention. All patients with previous endoscopy evaluation were followed up for EGD procedure after 6 months.

**TABLE 1:** HCC patients with gastroesophageal varices.

Sex/age	Liver disease etiology	Co‐morbidities	BCLC stage	Portal vein thrombosis	Cirrhotic (Y/N)	Child Pugh score	OGD	Combination therapy (Y/N)	Cycles	Patient's outcome	Patients' hematological results before treatment	Patients' hematological results after treatment
F/65	HBV	No	B	No	Y	B	Large GOV	N	4	m‐RECIST Partial Response	Hb: 11.1 Tt: 57 000	Hb: 11.1 Tt: 61 000
M/60	non‐Viral	No	C	Yes	Y	A	Small EV	Y (EBRT)	2	Not assessed yet	Hb: 14.3 Tt: 123 000	Hb: 11.5 Tt: 126 000
M/50	MAFLD	No	A	No	Y	B	Large EV	N	1	N/A	Hb: 14.5 Tt: 54 000	Hb: 14.6 Tt: 59 000
M/73	non‐Viral	No	C	Yes	Y	A	Large EV	N	6	m‐RECIST Partial Response	Hb: 13.6 Tt: 219 000	Hb: 13.9 Tt: 241 000
M/80	non‐Viral	DM	A	No	Y	A	Large EV	Y (RFA)	9	m‐RECIST Progression Disease	Hb: 14.4 Tt: 157 000	Hb: 14.4 Tt: 157 000
M/60	non‐Viral	DM	C	Yes	Y	A	Large EV	N	4	N/A	Hb: 14.3 Tt: 123 000	Hb: 11.5 Tt: 126 000
M/71	non‐Viral	DM	A	No	Y	A	Large EV	N	6	m‐RECIST Partial Response	Hb: 15.1 Tt: 110 000	Hb: 14.9 Tt: 128 000

Abbreviations: DM: diabetes mellitus; EBRT: external beam radiotherapy; EV: esophageal varices; GOV: gastroesophageal varices; Hb: hemoglobin levels; HBV: hepatitis B virus; m‐RECIST: modified response evaluation criteria in solid tumors; MAFLD: metabolic dysfunction fatty liver disease; OGD: oesophagogastroduodenoscopy; RFA: radiofrequency ablation; Tt: thrombocyte levels.

## 3 Discussion

Until now, there has been lacking data regarding IT used in HCC patients with the presence of significant or large gastroesophageal varices. Based on our real life‐experience case series study, it showed the safety of standard IT which were given in HCC patients with large GOV as soon as the varices were managed endoscopically. No bleeding was recorded based on our database. According to the guideline released by The American Society of Clinical Oncology (ASCO), combination of atezolizumab and bevacizumab is recommended as the first‐line therapy for patients with advanced HCC, especially for those with Child‐Pugh class A liver function and Eastern Cooperative Oncology Group (ECOG) performance status of 0–1 [9]. It is worth noting that the guideline recommends for a management of existing esophageal varices prior to the treatment due to the risk of bleeding [9]. The Baveno VII consensus for portal hypertension recommends the usage of non‐selective beta blockers (NSBB) to compensate for clinically significant portal hypertension in cirrhotic patients, marked by gastroesophageal varices and ascites [10]. NSBB prophylaxis is indicated if varices are present before or after the combination therapy of atezolizumab and bevacizumab [10]. In the presence of large EV as well as GV, it has been recommended to postpone the IT at least 1 week after endoscopic treatment due to the bleeding control or possibility of post banding bleeding ulcer. In our case series study, we successfully managed the large varices using EUS procedure for evaluation prior to endoscopic band ligation, as well as the therapeutic choice to prevent the bleeding during IT. Currently, EUS has been used widely for managing large GOV [11]. In our case series study, one patient with large GOV has been managed with EUS‐guided cyanoacrylate (CYA) injection. Another patient with Child‐Pugh B score underwent EUS evaluation before endoscopic band ligation due to recurrent EV with history of hematemesis. This patient underwent EUS‐guided portal pressure measurement (PPGM). This method can be a novel procedure for primary as well as secondary prophylaxis of AVB, which has been proven to be accurately for delivery of therapeutic agent and flow eradication in the varix, and effectively increasing the bleeding‐free survival of patients with minimal AEs compared to patients who received standard care [11].

Liver cirrhosis is a clinico‐pathological condition towards the development of esophageal varices, with portal hypertension being prevalent in roughly 50% of patients [12]. The cirrhotic liver is unable to produce a sufficient amount of nitric oxide (NO) to counter the active vasoconstriction in patients with cirrhosis, increasing the intrahepatic vascular pressure and exacerbating portal hypertension [12]. In addition, vasodilation of the splanchnic organs occurs in cirrhotic patients and thus increases the portal venous inflow [12]. This, together with intrahepatic vasoconstriction, results in a greater mismatch in blood flow and pressure regulation, worsening portal hypertension [12]. However, it is important to consider that based on a cohort study conducted by Johnson et al., 72% of patients that had developed HCC were indicated of having liver cirrhosis with Fib‐4 values above 3.25 before the diagnosis of HCC [13]. However, out of all HCC patients that progressed through cirrhosis, only 7% were HBV‐related, with the rest being related to either HCV (86%) or other causes [13]. In reflection to our case series, it differs in the way that all our patients had cirrhosis while none had the etiology of HCV [13]. Out of the seven patients presenting with varices, five patients stemmed from non‐viral etiologies possible due to metabolic condition, one patient from HBV, and one patient clearly diagnosed with MAFLD [13].

The use of atezolizumab and bevacizumab therapy for HCC has been related to high risk of bleeding [5]. Atezolizumab is a selective PD‐1 receptor antagonist on T cells and PD‐L1 protein on tumor cells, disturbing the inhibitory process of T cells, thus allowing them to continue their function in attacking tumor cells [5]. On the other hand, Bevacizumab acts as a monoclonal antibody that inhibits the vascular endothelial growth factor (VEGF), resulting in the obstruction of angiogenesis and hindering the growth of tumor cells [6]. As a molecular targeted agent (MTA), Bevacizumab not only inhibits vascularization of tumor cells, but also that of healthy organs, namely the thyroid, liver, pituitary gland, adrenal gland, and the gastrointestinal tract [14]. This inadvertently results in an increased manifestation of adverse events (AEs) [14]. The inhibition of NO as one of the mechanisms of an anti‐VEGF antibody leads to a prevention of vasodilation, which in turn increases the hepatic vascular tone and raises portal pressure—an event that responsibly causes varices [15]. Additionally, the inhibition of VEGF could subsequently decrease the capacity of endothelial regeneration [16]. As a result, a decrease of capillary bed density of the liver can manifest and cause pressure in the reticuloendothelial system to grow, leading to exacerbation of varicose veins [16]. Other than that, it also triggers a chain of events which alter vascular integrity and increase the risk of thromboembolism due to the exposure of coagulant‐activating phospholipids of the luminal plasma membrane [16]. If venous thromboembolism (VTE) clots a portal vein and causes portal vein thrombosis (PVT), blood pressure may be elevated and results in portal hypertension, which has been known as one of the causes for varices [17].

In our case series, all patients did not develop any bleeding despite starting the IT less than a week after receiving the endoscopic treatment. Our findings show that Atezolizumab and Bevacizumab combination therapy did not show a higher risk of variceal bleeding in HCC patients with portal hypertension given proper treatment and prophylaxis for gastroesophageal varices prior to IT [18]. The limitation of this study is the small sample size. However, these findings adhere to the various guidelines that the IMbrave150 trial has influenced in making said combination therapy the first‐line therapy in patients with unresectable or advanced HCC, with the trial only accepting patients with treated esophageal or gastric varices to reduce the probability of fatal bleeding during future treatment [18]. In conclusion, these cases demonstrate the potential safety of EUS‐guided endoscopic management before immunotherapy in HCC patients with varices.

## Funding

The authors have nothing to report.

## Ethics Statement

Before the data collection process, an ethical review was done by the Ethics Committee of the Faculty of Medicine, Pelita Harapan University with the certificate number: 221/K‐LKJ/ETIK/VI/2025.

## Consent

Informed consent from the participants was obtained.

## Conflicts of Interest

The authors declare no conflicts of interest.

## Data Availability

The data that support the findings of this study are available on request from the corresponding author. The data are not publicly available due to privacy or ethical restrictions.
